# Identification of genetic suppressors for a BSCL2 lipodystrophy pathogenic variant in *Caenorhabditis elegans*

**DOI:** 10.1242/dmm.050524

**Published:** 2024-04-16

**Authors:** Xiaofei Bai, Harold E. Smith, Andy Golden

**Affiliations:** ^1^ Department of Biology, University of Florida, Gainesville, FL 32610, USA; ^2^ Genetics Institute, University of Florida, Gainesville, FL 32610, USA; ^3^ National Institute of Diabetes and Digestive and Kidney Diseases, National Institutes of Health, Bethesda, MD 20892, USA

**Keywords:** *Caenorhabditis elegans*, CRISPR/Cas9, Genetic suppressor, LMBR-1, Lipid droplet, Seipin

## Abstract

Seipin (BSCL2), a conserved endoplasmic reticulum protein, plays a critical role in lipid droplet (LD) biogenesis and in regulating LD morphology, pathogenic variants of which are associated with Berardinelli–Seip congenital generalized lipodystrophy type 2 (BSCL2). To model BSCL2 disease, we generated an orthologous *BSCL2* variant, *seip-1(A185P)*, in *Caenorhabditis elegans*. In this study, we conducted an unbiased chemical mutagenesis screen to identify genetic suppressors that restore embryonic viability in the *seip-1(A185P)* mutant background. A total of five suppressor lines were isolated and recovered from the screen. The defective phenotypes of *seip-1(A185P)*, including embryonic lethality and impaired eggshell formation, were significantly suppressed in each suppressor line. Two of the five suppressor lines also alleviated the enlarged LDs in the oocytes. We then mapped a suppressor candidate gene, *lmbr-1*, which is an ortholog of human limb development membrane protein 1 (*LMBR1*). The CRISPR/Cas9 edited *lmbr-1* suppressor alleles, *lmbr-1(S647F)* and *lmbr-1(P314L)*, both significantly suppressed embryonic lethality and defective eggshell formation in the *seip-1(A185P)* background. The newly identified suppressor lines offer valuable insights into potential genetic interactors and pathways that may regulate seipin in the lipodystrophy model.

## Teaser

**Summary:** In *Caenorhabditis elegans*, the endoplasmic reticulum protein LMBR-1 serves as a genetic suppressor of the specific seipin mutation associated with congenital generalized lipodystrophy.

**Research Simplified**

Berardinelli-Seip congenital lipodystrophy 2 (BSCL2) is a rare condition that is characterised by loss or abnormal distribution of fat tissues in humans. BSCL2 is caused by gene variants that alter a protein called Seipin. Seipin regulates the size and shape of lipid droplets in cells that are important for efficient metabolism and maintenance of healthy physiology. Gaining a better understanding of what other gene variants affect BSCL2 and how they interact with altered Seipin protein can help researchers advance therapeutic possibilities for BSCL2.

First, the authors of this study introduced altered Seipin protein into laboratory nematode model *Caenorhabditis elegans* (worms) because humans and worms share strong resemblance in the way lipids are metabolised. This had lethal effects in worm embryos, marked by partially broken eggshells. In these worms, the authors then identified an additional gene called *lmbr1*, also present in humans, that when mutated reversed the effects of altered Seipin and suppressed BSCL2-associated lethality by significantly restoring defective eggshells.

This work uncovered a previously unknown gene that may regulate Seipin and modify how BSCL2 develops. Further investigation into these genes can help researchers develop potential therapeutic targets against BSCL2.

## INTRODUCTION

Lipid droplets (LDs) are cellular energy reservoirs to maintain fat homeostasis and balance, ensuring proper lipid metabolism and healthy physiology (Fei et al., 2008). Failure to maintain the size and morphology of LDs can lead to many diseases and disorders, including obesity, cancer, lipodystrophy and neurodegenerative disorders. (Onal et al., 2017; Pressly et al., 2022; Teixeira et al., 2021; Lim et al., 2021; Patni and Garg, 2015; Zadoorian et al., 2023) Seipin is one of the primary LD scaffold proteins to govern LD size, and may also regulate lipid biosynthesis and sorting to promote LD synthesis at the endoplasmic reticulum (ER). (Szymanski et al., 2007; Su et al., 2019; Wang et al., 2016). Clinical studies have identified multiple seipin pathogenic variants in patients with Berardinelli–Seip congenital lipodystrophy 2 (BSCL2), Silver syndrome and teratozoospermia syndrome (Magre et al., 2001; Agarwal and Garg, 2004; Windpassinger et al., 2004; Jiang et al., 2014). Therefore, understanding the molecular and cellular mechanisms governing LD formation and the nature of LD scaffold proteins, such as seipin, are critical to developing therapeutic strategies for LD-associated diseases.

Proper lipid metabolism and transfer are critical to oogenesis, fertilization, embryogenesis and other physiological processes, such as lifespan and locomotion, in *Caenorhabditis elegans*. Two lipid reservoirs, yolk granules and LDs, were identified in *C. elegans* oocytes and embryos as regulating lipid transfer and metabolism. However, the molecular and cellular mechanisms of how these two lipid structures contribute to healthy physiology remain unexplored. Our recent study established a *BSCL2* (seipin) disease model for understanding seipin-associated disorders *in vivo* (Bai et al., 2020a). Three *seip-1* null alleles and an orthologous BSCL2 pathogenic variant allele, *seip-1(A185P)*, caused severe embryonic lethality and abnormally enlarged LDs in *C. elegans* oocytes and embryos (Bai et al., 2020a). The embryonic lethality in the seipin mutants was correlatively linked to the impaired permeability barrier of the eggshell, a lipid-enriched extracellular matrix layer surrounding the embryo. Lipidomic studies showed that the C20 polyunsaturated fatty acid content was significantly reduced in the seipin mutants. The dietary supplementation of two types of linoleic acids restored embryonic lethality and extracellular eggshell formation in the seipin mutants. Intriguingly, the supplementation of the linoleic acids failed to rescue the abnormal/enlarged LD size and instead led to excessively enlarged LDs in oocytes and embryos (Bai et al., 2020a), suggesting that proper eggshell and LD formation were independently regulated by seipin. However, the exact contributions of seipin during these two cellular events remain obscure.

The nematode model *C. elegans* has become an emerging system for understanding lipid and lipid metabolism diseases (Shi et al., 2016; Yue et al., 2021). At least ten lipodystrophy-associated genes have been identified, including phosphate acetyltransferase (*AGPAT2*), phosphoinositide-dependent serine-threonine protein kinase (*AKT2*), seipin (*BSCL2*), caveolin 1 (*CAV1*) and perilipin 1 (*PLIN1*) (Kuivenhoven and Hegele, 2014). The conserved orthologs of all these lipodystrophy-associated genes are found in the *C. elegans* genome. Disturbing expression of the *C. elegans* orthologous genes led to various phenotypes, including embryonic lethality, sterility, lifespan alterations and locomotion defects (Bai et al., 2020a; Hertweck et al., 2004; Scheel et al., 1999; Chughtai et al., 2015). These easily scorable phenotypes allowed geneticists to conduct forward genetic screens to identify previously unidentified genetic modifiers for LD biogenesis and lipid signaling pathways (Wu et al., 2018; Ruiz et al., 2018; Li et al., 2016; Zhu et al., 2022). The development of rapid mapping strategies using molecular inversion probes (MIP-MAP; Mok et al., 2017) combined with high-throughput whole-genome sequencing provide a fast and cost-effective method for pinpointing candidate mutations in a mutagenized genetic model.

To explore more specific roles that seipin plays during embryonic and LD biosynthesis, we focused on identifying previously unknown genetic determinants and pathways. Taking advantage of the embryonic lethality in the *seip-1(A185P)* mutant as a readout, we conducted an unbiased forward genetic screen to identify genetic modifiers that restore embryonic viability in the *seip-1(A185P)* mutant. Using MIP-MAP genomic mapping and the whole-genome sequencing technique, we identified two independent missense alleles of a target suppressor gene *R05D3.2*, which we renamed *lmbr-1*. We then generated those two putative suppressor alleles of *lmbr-1*, *lmbr-1(S647F)* and *lmbr-1(P314L)*, in the wild-type background. The *lmbr-1* missense mutants were then crossed back into the *seip-1(A185P)* mutant to assess their suppression. The homozygous *lmbr-1(S647F)* and *lmbr-1(P314L)* alleles significantly suppressed embryonic lethality and eggshell formation defects caused by *seip-1(A185P)*. *lmbr-1(S647F)* did not alleviate the enlarged LD size caused by *seip-1(A185P)*, whereas the other allele, *lmbr-1(P314L)*, partially suppressed enlarged LD size in the *seip-1(A185P)* background. In summary, we describe the results of the mutagenesis screen designed to identify regulators that genetically interact with a seipin BSCL2 pathogenic variant*.* The discovery of new suppressor candidates will shed light on the molecular mechanisms contributing to seipin-associated lipodystrophy and related physiological disorders.

## RESULTS

### BSCL2 pathogenic variant impaired eggshell formation and caused enlarged LD size but did not alter the sub-cellular localization of SEIP-1::mScarlet

**Fig. 1.**
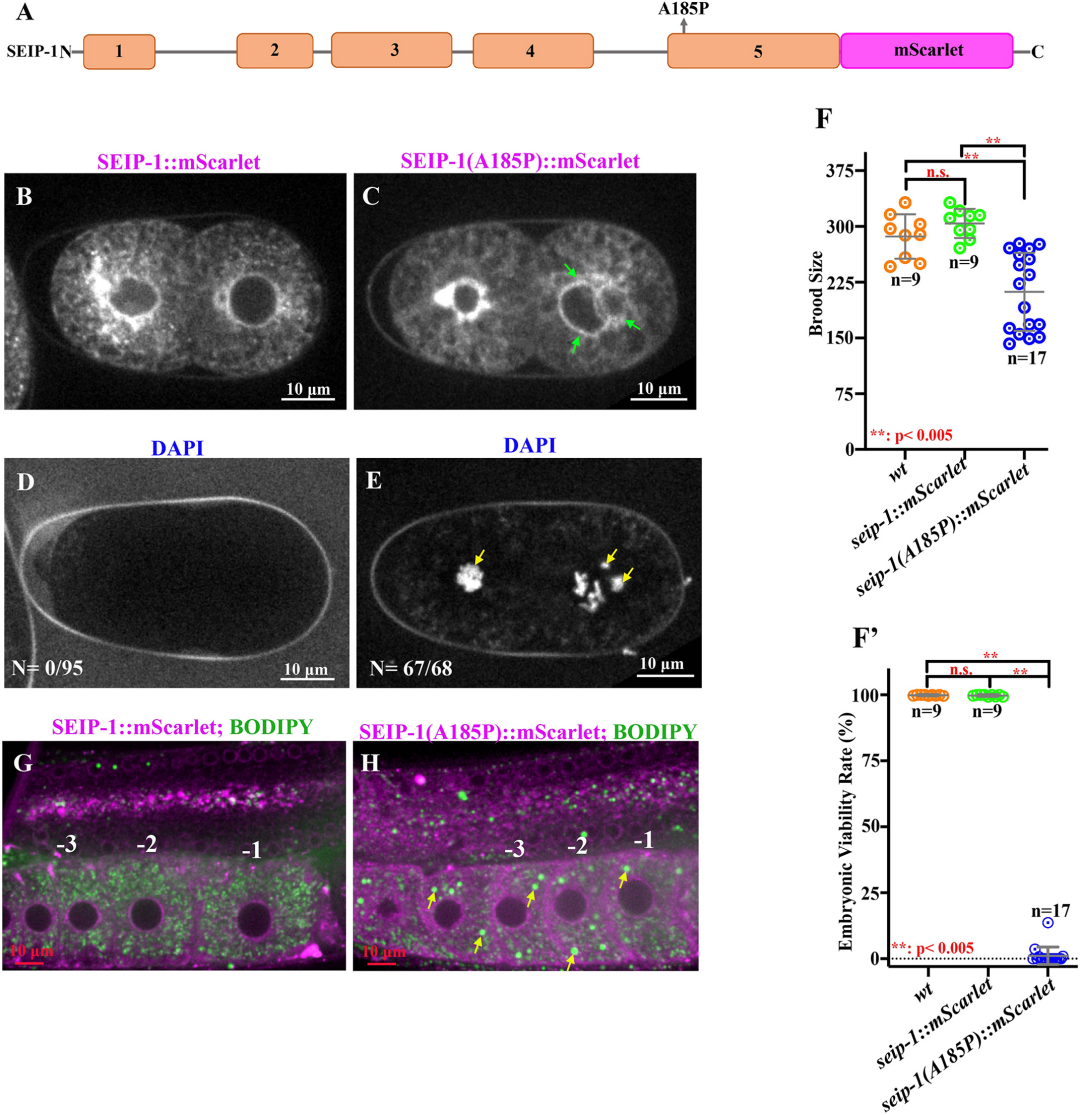
**Functional analysis of the A185P mutation in a *seip-1::mScarlet* reporter gene.** (A) Schematic diagram of *seip-1(A185P)::mScarlet*. (B,C) Visualization of mScarlet fluorescence to assess SEIP-1 localization. Green arrows indicate the presence of multiple nuclei in the posterior cell. (D,E) DAPI staining of embryos to assess eggshell permeability. Yellow arrows indicate the presence of stained chromatin. (F,F′) Brood size and embryonic viability of the mScarlet reporter genes. ns, not significant; **P<0.005 (one-way ANOVA with post hoc test). (G) SEIP-1 localization and lipid droplets in the adult germline. Representative images of animals containing the *seip-1::mScarlet* (G, magenta) or *seip-1(A185P)::mScarlet* (H, magenta) reporter, as visualized using mScarlet protein fluorescence (G,H, magenta), and the lipid dye BODIPY (G,H, green). Yellow arrows indicate the presence of enlarged lipid droplets. Oocytes are numbered conventionally (−1, −2 and −3) relative to the spermatheca, which is the site of fertilization. Scale bars: 10 μm. Each experiment was repeated at least three times. *n* values are indicated in the graphs.

Previously, we established a BSCL2 disease model by generating an orthologous pathogenic variant, *seip-1(A185P)*, in *C. elegans* (Bai et al., 2020a). This corresponds to the orthologous alanine 212 in the human *BSCL2* gene. Using this disease model, we could functionally characterize the *seip-1(A185P)* allele *in vivo*. *seip-1(A185P)* caused penetrant embryonic lethality, defective eggshell formation and abnormally enlarged LDs (Bai et al., 2020a). To determine whether this missense mutation disrupts the temporal and spatial expression pattern of SEIP-1, we generated the *seip-1(A185P)* allele by CRISPR/Cas9 genome editing into an endogenously tagged red fluorescent reporter strain, *seip-1::mScarlet* (Fig. 1A), which we designated *seip-1(A185P)::mScarlet*.

Embryonic localization of SEIP-1(A185P)::mScarlet was similar to the wild-type reporter, with enrichment around the nuclear envelope (Fig. 1B,C). This enrichment corresponds to the ER, as SEIP-1::mScarlet was shown previously to co-localize with the ER marker SP12 (Magre et al., 2001). However, the cytosolic distribution was more diffuse and less punctate in the *seip-1(A185P)::mScarlet* mutant compared with the wild type, suggesting a defect in ER structure or the association of seipin with the ER. The most apparent difference was the presence of multiple nuclei in the *seip-1(A185P)::mScarlet* strain (Fig. 1C, green arrows). This defect has not been reported previously and likely contributes to the embryonic lethality observed in the *seip-1(A185P)* strain. *seip-1::mScarlet* has previously been shown to express in additional *C. elegans* tissues, including the pharynx and epidermis, where the LDs are enriched (Bai et al., 2020a; Cao et al., 2019); similar localization patterns were found in the *seip-1(A185P)::mScarlet* mutant (Fig. S1). Imaging of the adult germline (see following) also revealed that *seip-1(A185P)::mScarlet* distribution was normal (Fig. 1G,H). Taken together, results from the fluorescent reporter genes suggest that the *seip-1(A185P)* mutation may compromise protein function instead of altering the trafficking and cellular localization of SEIP-1 *in vivo*.

We performed a variety of functional assays to test this hypothesis. We used DAPI, a small molecule that can penetrate into embryos with an impaired eggshell permeability barrier, to assess eggshell integrity in the *seip-1(A185P)::mScarlet* mutant. In the *seip-1::mScarlet* control strain, no DAPI staining was detected in the cytosol, demonstrating that knock-in of *mScarlet* at the endogenous loci of *seip-1* did not disrupt eggshell integrity (DAPI staining found in 0 out of 95 embryos) (Fig. 1D). In contrast, zygotic chromatin labeled by DAPI was frequently observed in the fertilized embryos of the *seip-1(A185P)::mScarlet* mutant (67 out of 68 embryos), which is indicative of impaired eggshell formation (Fig. 1E); we also observed multiple chromatin bodies (Fig. 1E, yellow arrows) associated with the multi-nucleate phenotype, which is a previously unreported observation in the *seip-1* mutants. Next, we assessed embryonic viability, as previous work indicated that the wild-type *seip-1::mScarlet* reporter did not affect survival (Bai et al., 2020a); in contrast, we found that *seip-1(A185P)::mScarlet* exhibited reduced brood size and embryonic lethality comparable with the *seip-1(A185P)* allele (Fig. 1F,F′). Finally, we examined LD size by staining animals with a lipophilic fluorescent probe BODIPY 493/503 in both *seip-1::mScarlet* control (Fig. 1G) and the *seip-1(A185P)::mScarlet* mutant (Fig. 1H). Enlarged LDs were observed in the oocytes on the proximal side of the gonad, which is adjacent to the spermatheca of the *seip-1(A185P)::mScarlet* animals (Fig. 1H). The defective phenotypes in the *seip-1(A185P)::mScarlet* mutant are consistent with other *seip-1* mutants characterized in the previous study (Bai et al., 2020a). We conclude that the primary impact of the A185P mutation is on protein function rather than localization, although a modest effect on the latter cannot be ruled out.

### EMS-based forward genetic screen to identify suppressors of *seip-1(A185P)*

**Fig. 2.**
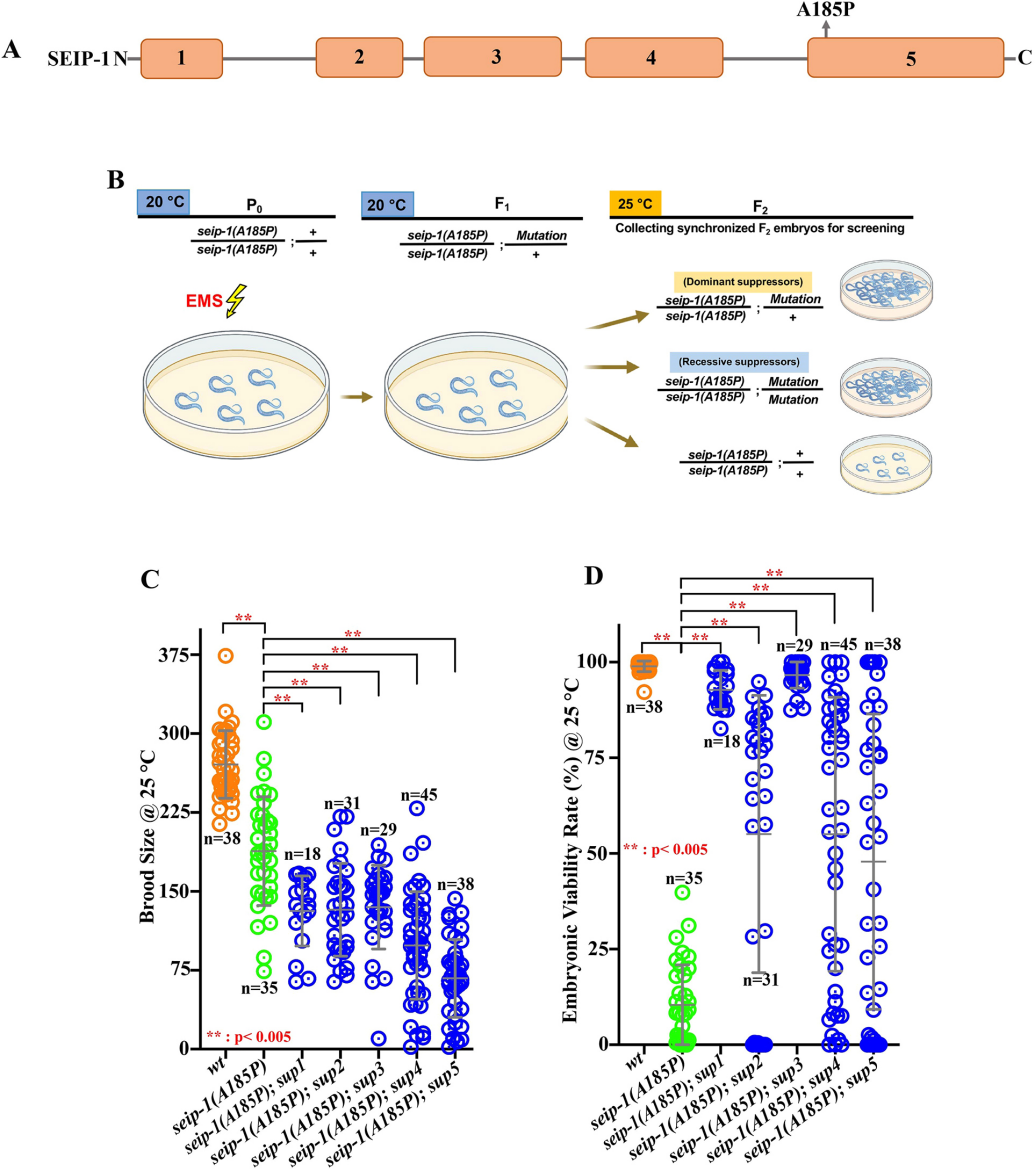
**Forward genetic screen for suppressors of the embryonic lethality of a seipin patient-specific allele.** (A) Diagram of the location of the *seip-1(A185P)* mutation in the *seip-1* gene. (B) Strategy to identify genetic modifiers that restored embryonic viability in the *seip-1(A185P)* mutant. Created with BioRender.com. (C) Total brood size of each isolated suppressor line, wild type and *seip-1(A185P)* mutant over 60 h post mid-L4. (D) The percentage of embryonic viability was significantly restored in each suppressor line when compared with *seip-1(A185P)* mutant only. Data are mean±s.d. Statistical significance was determined using one-way ANOVA with Tukey's post hoc test, **P<0.005. Each experiment was repeated at least three times. *n* values are indicated in the graphs.

To identify novel genetic modifiers that regulate seipin function, we conducted an ethyl methane sulfonate (EMS)-based forward genetic screen with the *seip-1(A185P)* mutant*.* The *seip-1(A185P)* allele caused a significantly reduced brood size, which includes both unhatched/dead embryos and hatched larvae, and penetrant embryonic lethality (Fig. 2A-D) (Bai et al., 2020a). During the course of screen optimization, we determined that these defects were enhanced at a higher temperature (25°C) and that the embryonic lethality in *seip-1(A185P)* worsened when the animals were maintained at 25°C for one to two generations. Therefore, we performed the mutagenesis screen under this optimized temperature to isolate and recover suppressor lines of *seip-1(A185P)* that restored embryonic viability and produced viable larvae (Fig. 2B). The screen yielded a total of five independent suppressor lines from ∼64,000 haploid *C. elegans* genomes (∼32,000 mutagenized F1s). To generate the homozygous suppressor lines, embryonic lethality in each line was assessed for at least two generations, testing more than 20 animals per generation. Only the candidate lines displaying consistently elevated viability rates in each tested animal were collected and identified as the homozygous suppressor lines. Sanger sequencing confirmed the *seip-1(A185P)* allele in each suppressor line. The embryonic viability rate in each suppressor line was significantly increased when compared with *seip-1(A185P)* allele alone (Fig. 2D). Yet, the brood size in each suppressor line was reduced (Fig. 2C). Overall, our findings suggest that homozygous suppressor variants might alleviate the embryonic lethality caused by the *seip-1(A185P)* mutant. However, these variants could potentially have additional impacts on reproduction, such as causing a reduction in brood size. It is also plausible that other essential mutations in the suppressor background could contribute to the observed sub-fertility.

### The defective eggshell formation was significantly restored in each suppressor line

**Fig. 3.**
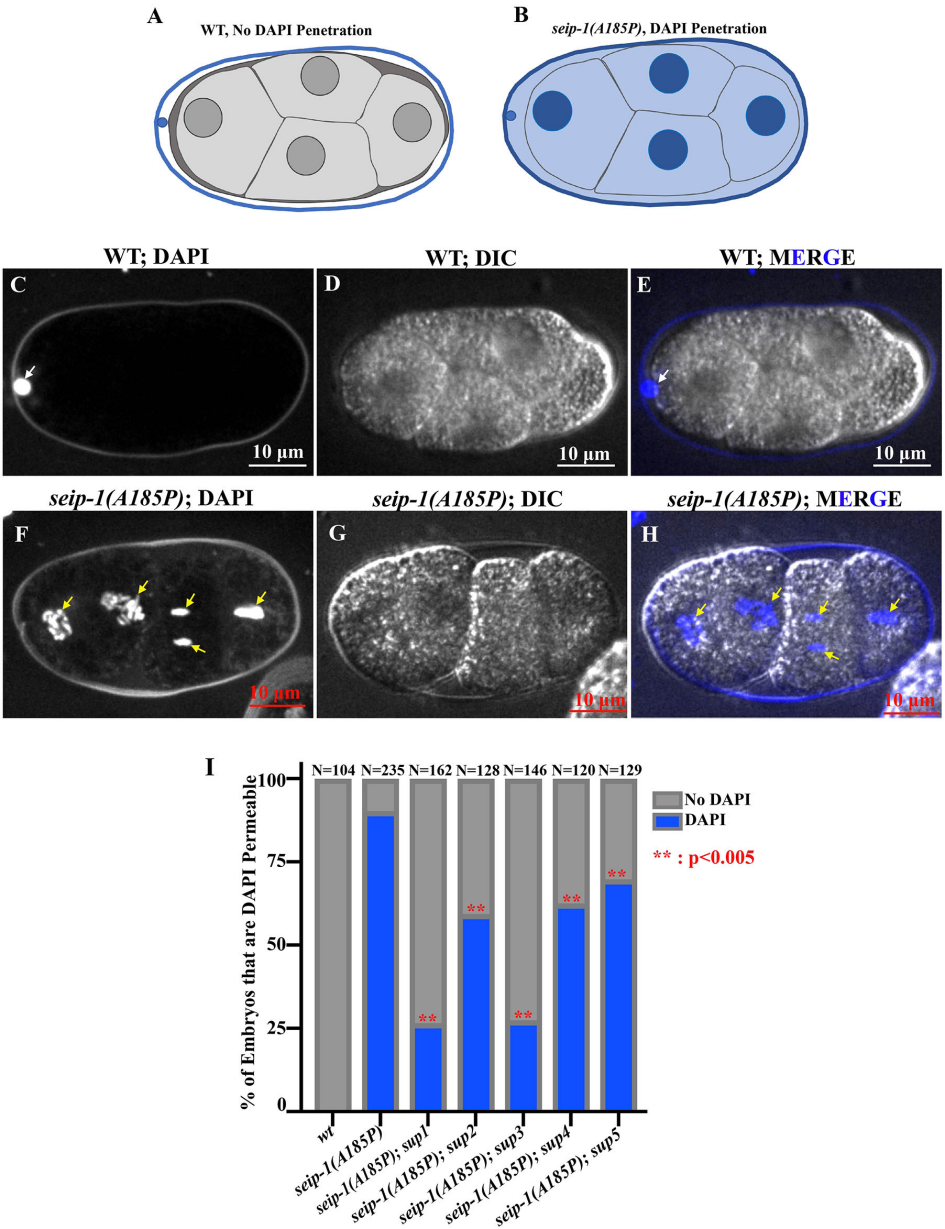
**Defective eggshell formation was restored in each suppressor line.** (A,B) Schematic diagram of DAPI staining assay in *C. elegans* embryos to assess eggshell permeability. (C-H) Representative images of the DAPI staining assay showed only the first polar body stained in wild type (A,E, white arrows), whereas DAPI penetrated the eggshell to stain the zygotic chromatin in *seip-1(A185P)* mutants (F,H, yellow arrows). DIC images of embryos are shown in D and G. (I) Quantification of the embryos with DAPI staining in wild type, *seip-1(A185P)* only and all suppressor lines. Statistical significance was determined using a Chi-square test. **P<0.005. Each experiment was repeated at least three times. *n* values are indicated in each panel.

The previous study indicated that the embryonic lethality of the *seip-1(A185P)* mutant was correlatively linked with impaired eggshell (Bai et al., 2020a). To test whether the eggshell formation deficiency was restored in each suppressor line, we imaged the DAPI penetration in the early embryos. In the wild type, only the first polar body, which is located outside the permeability barrier (one layer of the eggshell) that blocks small-molecule penetration, was stained by DAPI (Fig. 3A,C-E). In the *seip-1(A185P)* mutant, DAPI could penetrate the eggshell and stain the zygotic chromatin, indicating the defective permeability barrier (Fig. 3B,F-H). Using the DAPI imaging protocol, we tested over a hundred early embryos of each suppressor line. We found that DAPI penetration in each tested suppressor line was significantly reduced compared with that in the *seip-1(A185P)* mutant (Fig. 3I). These observations suggested that the putative modifiers in the suppressor lines restore the proper formation of the permeability barrier. Not surprisingly, the suppressor lines *seip-1(A185P); sup1* and *seip-1(A185P); sup3*, which displayed the least DAPI penetration, correlated with the highest levels of embryonic viability (Figs 2D and 3I), supporting our proposed hypothesis that the embryonic lethality in the *seip-1(A185P)* mutant background is linked with the proper eggshell formation.

### Abnormal enlarged LD size was not correlatively rescued in each suppressor line of *seip-1(A185P)*

**Fig. 4.**
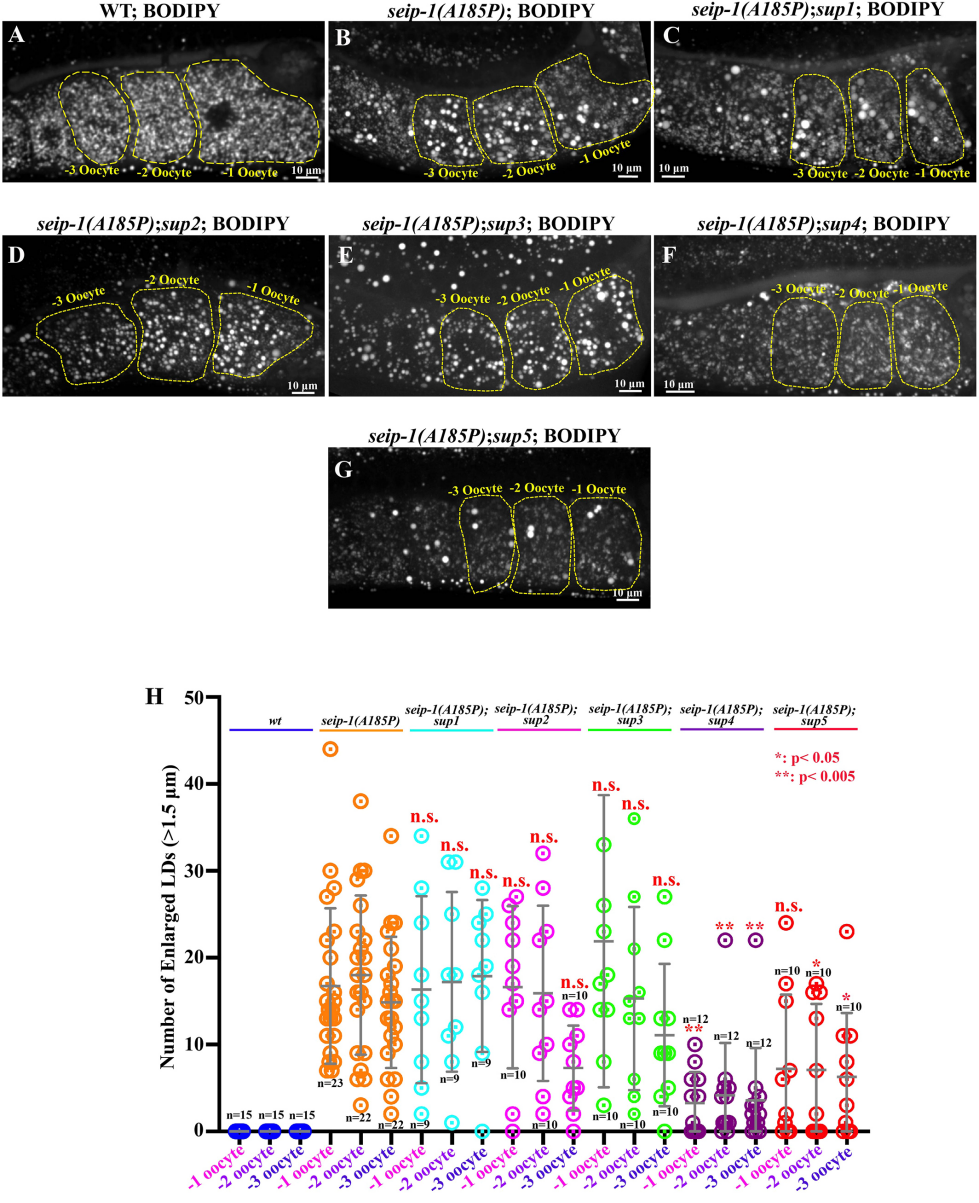
**Enlarged lipid droplets were not correlatively restored in the suppressor lines.** (A-G) BODIPY-stained lipid droplets (LDs) in the −1 to −3 oocytes of wild type (A), *seip-1(A185P)* only (B) and all five suppressor lines (C-G). (H) Quantification of the enlarged LDs (diameter >1.5 μm) inside the −1 to −3 oocytes of each stained genotype. Data are mean±s.d. Statistical significance was determined using one-way ANOVA with Tukey's post hoc test. *P<0.05; **P<0.005. Scale bars: 10 μm. Each experiment was repeated for at least three times. *n* values are indicated in each panel.

The canonical function of seipin is to maintain LD size and biosynthesis. We found that irregularly enlarged LDs were observed in the oocytes and embryos of the *seip-1(A185P)* mutant compared with wild type (Bai et al., 2020a) (Fig. 4A,B,H). To test whether the abnormal LD size was alleviated in the suppressor lines, we imaged the oocytes in which LDs were enriched by staining with a lipophilic BODIPY neutral fluorophore that binds to neutral lipids (Fig. 4A-G). The parameter of LD size was defined and quantified by measuring the number of LDs with diameters larger than 1.5 μm in the −1 to −3 oocytes, as described in the previous study (Bai et al., 2020a). In wild type, the average LD size was less than 0.5 μm (Fig. 4A,H), but more than 20 enlarged LDs (>1.5 μm) were readily stained in the −1 to −3 oocytes in the *seip-1(A185P)* mutant (Fig. 4B,H). The enlarged LD size (>1.5 μm) was only alleviated in two of five suppressor lines: *seip-1(A185P); sup4* and *seip-1(A185P); sup5* (Fig. 4F-G,H)*.* These two suppressor lines displayed the most rescued LD phenotypes. Furthermore, they had the highest levels of DAPI penetration and embryonic lethality (Figs 2D and 3I), suggesting that genetic modifiers in each suppressor line may function somewhat independently in regulating eggshell formation, embryonic viability and the maintenance of proper LD size in the oocyte. These observations are consistent with our previous observation that dietary supplementation of polyunsaturated fatty acids significantly restored embryonic lethality and defective eggshell formation but enhanced the abnormal enlarged LD size (Magre et al., 2001). This finding was also observed in a study indicating that the changes in phospholipid synthesis could suppress embryonic lethality but not affect LD size in a *seip-1* null mutant (Zhu et al., 2022). In summary, our data suggested that maintaining proper LD size primarily contributes to maintaining lipid balance and metabolism homeostasis, but may only play a limited role in orchestrating eggshell formation.

### MIP-MAP mapping and suppressor allele validation

**Fig. 5.**
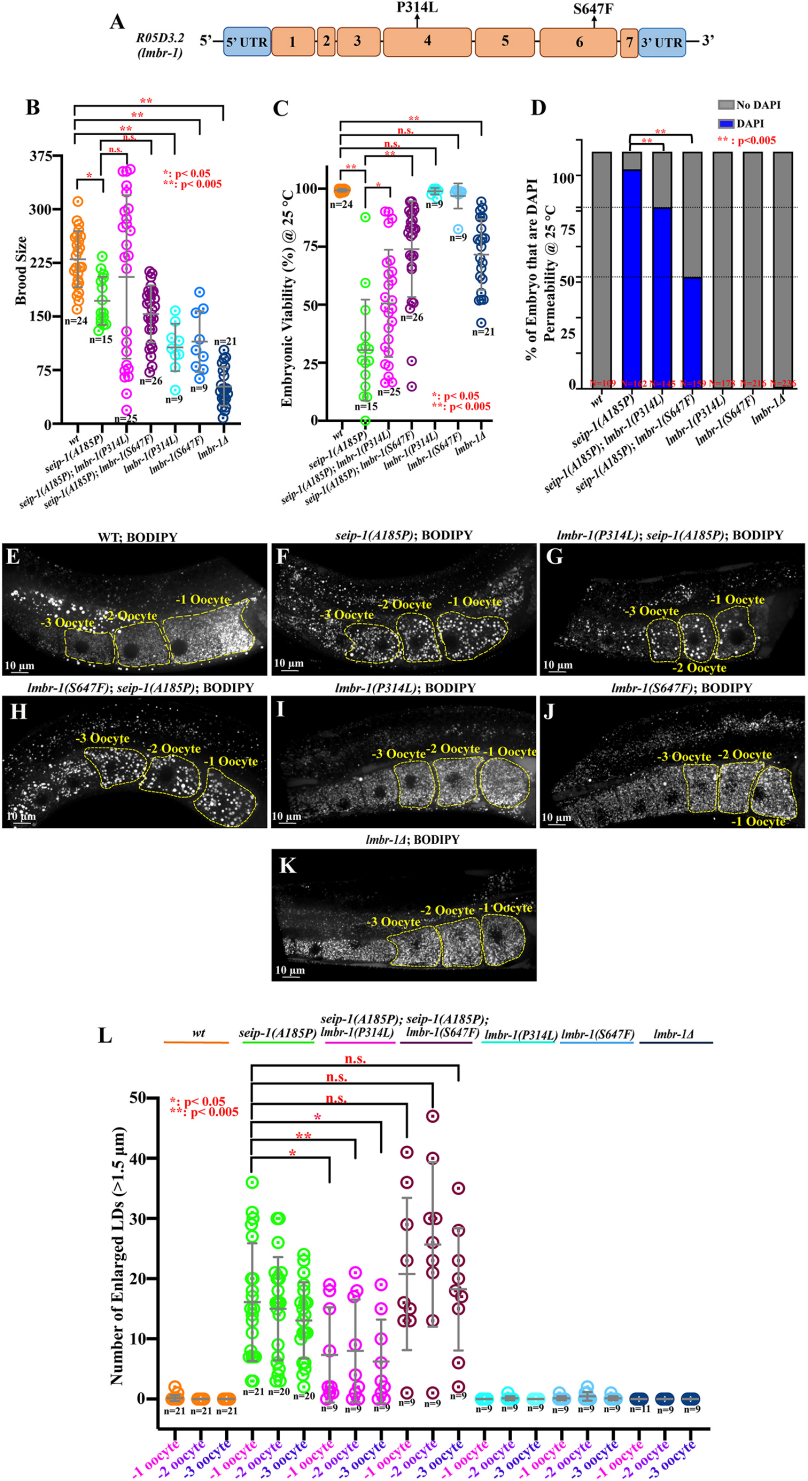
**Identification of the genetic modifiers by MIP-MAP genome mapping and whole-genome sequencing.** (A) Schematic diagram of two suppressor alleles of *lmbr-1* were generated in the wild-type background. (B,C) Total brood size and embryonic viability of each isolated wild type, *seip-1(A185P)*, *lmbr-1* suppressor alleles, *lmbr-1* null mutant, and double mutants of *seip-1(A185P)* and *lmbr-1* alleles. *P<0.05, **P<0.005 (χ^2^ test) (D) DAPI penetration in the early embryos of each tested mutant and wild type.**P<0.005 (χ^2^ test). (E-K) Representative images of the BODIPY-stained LDs in the −1 to −3 oocytes of wild type (E), *seip-1(A185P)* (F), *lmbr-1(P314L)* (I), *lmbr-1(S647F)* (J), *lmbr-1Δ* (K), and *lmbr-1(P314L); seip-1(A185P)* (G) and *lmbr-1(S647F); seip-1(A185P)* double mutants (H). (L) Quantification of the enlarged LDs (diameter >1.5 μm) in the indicated genotypes. *P<0.05, **P<0.005 (one-way ANOVA with Tukey's post hoc test). Each experiment was repeated for at least three times. *n* values are indicated in each panel.

Using a fast and high-throughput genomic mapping strategy that involves molecular inversion probes (MIP-MAP; Mok et al., 2017), we were able to identify the genetic modifiers in the suppressor line *seip-1; sup1* and *seip-1; sup4* (Fig. S2). We used a superficial wild-type strain, VC20019, with sufficient genomic diversity to provide∼1 Mb mapping resolution. To maintain the *seip-1(A185P)* allele during the mapping process, we generated *seip-1(A185P)* in the VC20019 background by CRISPR-mediated genome editing. After mating and propagation to select for animals that contained the suppressor, we constructed MIP-MAP and whole-genome libraries for next-generation sequencing. For two of the suppressor lines, *seip-1; sup1* and *seip-1; sup4*, we mapped the suppressors to overlapping genomic regions on chromosome III (*sup1*, 5.3-9.2 Mb, Fig. S2B; *sup-4*, 5.5-9.2 Mb, Fig. S2E). The mapping plots of the remaining suppressors either contained multiple gaps and peaks (*seip-1; sup3*) (Fig. S2D), indicative of multiple loci under selection, or no gaps (*seip-1; sup2* and *seip-1; sup5*) (Fig. S2C,F), consistent with dominant suppressors (Fig. S2). Therefore, we focused on suppressor lines *seip-1; sup1* and *seip-1; sup4.* After restricting the list of mutations to homozygous, protein-coding variants in the mapped regions and comparing the lists, we identified a single candidate modifier gene, *R05D3.2*, that contained different missense mutations in the two suppressor lines: *R05D3.2(S647F)* in *seip-1; sup1* and *R05D3.2(P314L)* in *seip-1; sup4 (*Fig. 5A). *R05D3.2* is an ortholog of human limb development membrane protein 1 (*LMBR1*) and we renamed the *C. elegans R05D3.2* to *lmbr-1*.

To validate *lmbr-1* as the suppressor candidate, we regenerated the two putative suppressor alleles of the *lmbr-1* gene, *lmbr-1(P314L)* and *lmbr-1(S647F)*, in the wild-type background by CRISPR/Cas9-mediated genome editing (Fig. 5A). Both *lmbr-1(P314L)* and *lmbr-1(S647F)* mutations alone caused reduced brood size compared with wild type (Fig. 5B); however, there were no embryonic lethality or eggshell formation defects observed in the *lmbr-1* missense mutants (Fig. 5C,D). To further characterize the role of *lmbr-1* in *C. elegans*, we also generated a null mutation, *lmbr-1Δ*, which bears a full-length deletion of the *lmbr-1* gene*.* Compared with the P314L and S647F alleles, the homozygous *lmbr-1Δ* mutant caused smaller brood size and ∼25% embryonic lethality (Fig. 5B,C); this result indicates that *lmbr-1* is critical to *C. elegans* reproduction and embryogenesis, and suggests that the missense mutations are not loss-of-function alleles. Additionally, we did not observe any eggshell defects in the *lmbr-1Δ* mutant (Fig. 5D), suggesting that the embryonic lethality caused by *lmbr-1Δ* is not associated with the integrity of eggshell. To further investigate the expression pattern of *lmbr-1* in *C. elegans*, we successfully knocked in a fluorescent reporter *gfp* at the N terminus loci of the *lmbr-1* gene. However, we could not detect any visible fluorescence signal of GFP::LMBR-1, likely due to the low expression level of *lmbr-1* in *C. elegans*.

We then crossed the newly generated *lmbr-1(P314L)* and *lmbr-1(S647F)* alleles into the *seip-1(A185P)* background to test their suppression in the context of embryonic lethality, eggshell formation and enlarged LD size. Both *lmbr-1(P314L)* and *lmbr-1(S647F)* significantly suppressed embryonic lethality and the defective eggshell formation but did not affect the brood size compared with the effects of *seip-1(A185P)* alone (Fig. 5B-D). Additionally, all *lmbr-1* mutants, including *lmbr-1(P314L)*, *lmbr-1(S647F)* and *lmbr-1Δ* alone did not affect LD size in the −1 to −3 oocytes compared with wild type (Fig. 5E,I-L). Intriguingly, the *lmbr-1(P314L)* allele partially suppressed the irregularly enlarged LD size in the −1 to −3 oocytes of the *seip-1(A185P)* mutant (Fig. 5F,G,L). This finding is consistent with the alleviated enlarged LDs phenotype in the original suppressor line *seip-1(A185P); sup4*, which contains the *lmbr-1(P314L)* allele. The other allele *lmbr-1(S647F)*, like the *seip-1(A185P); sup1* strain from which it was identified, did not affect the enlarged LDs compared with the *seip-1(A185P)* mutant (Fig. 5F,H,L). Overall, the CRISPR-edited *lmbr-1* alleles displayed identical suppression of embryonic lethality, defective eggshell formation and enlarged LD phenotypes that were observed in the original suppressor lines. Additionally, different *lmbr-1* suppressor alleles, such as P314*L* and S647F, may contribute independently to regulating embryonic lethality/defective eggshell integrity and enlarged LD size.

## DISCUSSION

The primary objective of the forward genetic screen in the orthologous lipodystrophy *seip-1(A185P)* mutant background was to uncover previously unreported genetic determinants and regulators of seipin. We sought to identify and characterize the genetic modifiers capable of restoring cellular and developmental deficiencies in the *seip-1(A185P)* mutant background. In the course of this study, we successfully isolated five distinct suppressor lines. Furthermore, we refined the MIP-MAP strategy by introducing the pathogenic variant into the mapping strain using CRISPR/Cas9 editing. This strategic innovation significantly streamlined the mapping process and reduced both labor and time required for identifying potential modifier candidates. During the trial screen, we identified two suppressor alleles, including *lmbr-1(P314L)* and *lmbr-1(S647F)*, within the newly implicated genetic determinant, *lmbr-1*. These alleles demonstrated a remarkable ability to suppress embryonic lethality and defective eggshell formation resulting from the *seip-1(A185P)* mutation.

### LMBR-1 and its suppression in the *seip-1(A185P)* mutant

The *C. elegans* gene *R05D3.2* is the only ortholog of human *LMBR1* and LMBR1-like membrane protein (*LMBR1L*). Both LMBR1 and LMBR1L exhibit high conservation, sharing 60% identity in their amino acid sequence. A distinguishing feature found among all LMBR1 proteins is the presence of multiple transmembrane segments. These LMBR1 proteins are predicted to express at the ER membrane, similar to seipin expression (Choi et al., 2019). Notably, the LMBR1L protein is also known as lipocalin 1-interacting membrane receptor (LIMR) (Hesselink and Findlay, 2013), which interacts with lipocalin 1, an extracellular protein responsible for binding hydrophobic ligands, fatty acids and phospholipids, thereby facilitating their internalization and subsequent degradation (Tsukamoto et al., 2009; Glasgow, 2021; Glasgow and Abduragimov, 2021).

Therefore, the *lmbr-1* suppressor mutations are plausible candidates to influence lipid transfer or lipid metabolism, potentially acting to restore the disturbed lipid balance within the *seip-1(A185P)* mutant. Consequently, these mutations may contribute to the suppression of embryonic lethality and other defective phenotypes. Further investigations will be directed toward a better understanding of the underlying molecular mechanism driving this suppression. In particular, we aim to characterize the intricate interplay between lipid metabolism and the functional coordination between seipin and *lmbr-1*.

In the scope of this study, we also characterized the functional contribution of *lmbr-1* by generating multiple mutants, including two missense suppressor alleles and a *lmbr-1* null mutant bearing a full-length deletion. All tested *lmbr-1* mutants displayed a reduction in brood size, and the null mutant exhibited about 30% of embryonic lethality. Interestingly, previous research has shed light on the role of LMBR1L as a pivotal player in cell signaling regulation. Specifically, it is known to modulate Wnt/catenin and BMP signaling pathways in contexts of lymphocyte development and *Drosophila* oogenesis (Cao et al., 2019; Glasgow and Abduragimov, 2021). Collectively, these findings underscore that *lmbr-1* may serve diverse functional roles that significantly contribute to the intricate dynamics of *C. elegans* reproduction and embryonic development. The observations also serve as an explanation for the reduced brood size in the original *seip-1* suppressor lines.

Finally, the CRISPR-generated *lmbr-1* mutations showed a relatively low level of suppression of DAPI penetration compared with the EMS-derived strains, suggesting that additional modifiers likely await discovery. However, we could not exclude the possibility that other cellular signaling pathways may be involved in regulating lipid droplet, embryogenesis and eggshell formation in the seipin mutants. *lmbr-1* suppressor alleles may also be involved in other signaling pathways to coordinate the suppression of the defects.

### Congenital lipodystrophy disease modeling and future direction

In this study, we observed the emergence of a multi-nucleate and defective nuclear envelope formation, which appears to play a pivotal role in the observed lethality of the *seip-1(A185P)* mutant. Furthermore, similar nuclear envelope phenotypes were also associated with lipodystrophies, such as partial lipodystrophy of the Dunnigan type (FPLD2) (Guenantin et al., 2014). Thus, cellular-level similarities could be relevant to human diseases, even though *C. elegans* lacks adipose tissues, which show significant phenotypes in human lipodystrophy conditions.

Our study also introduces an accessible *in vivo* system for investigating seipin in the *C. elegans* germline tissue, which is enriched in LDs and necessitates the transfer of lipids and fatty acids between somatic tissue during ovulation and fertilization. The mechanisms involving lipid transportation and metabolic regulation of these structures are likely conserved between humans and *C. elegans*. Moreover, the observed embryonic lethality in *seip-1(A185P)* mutants has allowed us to screen genetic antagonists specific to *seip-1* patient-specific alleles *in vivo*. For future studies, we intend to identify the currently unknown genetic modifiers in the other three suppressor lines using the alternative mapping crosses more suitable for polygenic or dominant mutations. Our suitable paradigm of embryonic lethality in seipin mutants should allow us to continue forward screens to identify molecular pathways or determinants to suppress or reverse the defects. The findings in our genetic study may provide insights for future therapeutic targets. A successful reproductive cycle is precisely coordinated by lipid or modified lipid signaling to fertilization, oocyte growth, meiotic progression and gonadal muscle contraction in *C. elegans*. The overarching theme of our study is to delineate the signaling mechanism of seipin in coordinating these physiological processes. Understanding these signaling pathways involving seipin should advance our understanding of lipid biology in other organisms, including humans.

## MATERIALS AND METHODS

### Strain maintenance

The strains used in this study were maintained on MYOB plates as previously described at 20°C or 25°C when screening the suppressors (Burns et al., 2006). Detailed information of the strains is as follows: N2 Bristol (wild-type); AG429, *seip-1(av169[A185P]) V*. CRISPR/Cas9 Edit; AG444, *seip-1(av169[seip-1::mScarlet]) V*. CRISPR/Cas9 Edit; AG666, *seip-1(A185P) V; MIP-MAP*. CRISPR/Cas9 Edit; AG685, *seip-1(av304[seip-1(A185P)::mScarlet]) V*. CRISPR/Cas9 Edit; AG670, *seip-1(av169[A185P]) V; sup1*; AG671, *seip-1(av169[A185P]) V; sup2*; AG686, *seip-1(av169[A185P]) V; sup3*; AG687, *seip-1(av169[A185P]) V; sup4*; AG688, *seip-1(av169[A185P]) V; sup5*; AG743, *lmbr-1(av288[P314L]) III*; AG746, *lmbr-1(av291[S647F]) III*; AG750, *lmbr-1(av293[lmbr-1Δ] III*; AG751, *seip-1(av294) V*; AG755, *seip-1(av294) V*; *lmbr-1(av288[P314L]) III*; and AG756, *seip-1(av294) V*; *lmbr-1(av291[S647F]) III.*

### EMS suppressor screen

*seip-1(A185P)* early L4 hermaphrodites were washed three times in M9 buffer and soaked in 48 mM ethyl methane sulfonate (EMS) solution for 4 h at room temperature. The EMS-treated animals were washed three times in M9 buffer and were then transferred to a fresh 100 mm MYOB plate with OP50 on one side. The animals were allowed to recover for up to 4 h before being picked to 100 mm MYOB plates with fresh OP50. Only the recovered animals that were able to crawl across the plates to the OP50 food were transferred to the fresh plates. A total of 15 MYOB plates with ten to 15 mid-L4 (P0s) on each were incubated at 20°C. Gravid F1 adult progeny (∼32,000) were collected for synchronizing the F2 population using hypochlorite treatment. The F2 embryos were shaken in a glass flask with M9 buffer overnight at 20°C, the hatched larvae were shifted to 25°C and their F3 progeny were screened for viable larvae. A total of five suppressor lines were isolated from the screen. The males of *seip-1(A185P^MM^)* were mated with the homozygous hermaphrodites of each suppressor line (Fig. S2A). We carefully pooled F2 progeny from the cross and allowed the F2 population to expand for over ten generations for MIP-MAP analysis, which is sufficient to distribute the MIP-MAP molecular probes into the suppressor line background and provides a high molecular resolution for identifying the mutation regions (Fig. S2A). Theoretically, we should be able to narrow down the target regions bearing the putative modifiers by reading the frequency of MIP-MAP probes. As the flanking regions of the modifiers originated from a wild-type background, the occurrence frequency of the MIP-MAP probes at the nearby loci would drop to nearly zero (Mok et al., 2017).

### Microscope and imaging analysis

For imaging SEIP-1::mScarlet and SEIP-1(A185P)::mScarlet expression, animals were immobilized on 7% agar pads with anesthetic (0.1% tricaine and 0.01% tetramisole in M9 buffer). Differential interference contrast (DIC) and mScarlet image acquisition were conducted using a Nikon 60×1.2 NA water objective with 1 μm *z*-step size; 20-25 *z-*planes were captured. The imaging was performed on a scanning-disk confocal system, including a Yokogawa CSU-X1 confocal scanner unit, a Nikon 60×1.2 NA water objective, and a Photometrics Prime 95B EMCCD camera. The images were analyzed using NIS imaging software (Nikon) and ImageJ/FIJI Bio-formats plugin (National Institutes of Health) (Linkert et al., 2010; Schindelin et al., 2012).

### DAPI staining of embryos

Gravid hermaphrodites were subject to three washes in M9 buffer and subsequently dissected using 23G×3/4″ needles. Embryos at various developmental stages were then transferred into a handing drop chamber that was pre-filled with blastomere culture medium (BCM), as described in the previous study (Bai and Bembenek, 2017). The BCM was prepared freshly, containing 10 μg/ml DAPI (BD Biosciences, BD Pharmingen DAPI solution, #564907) for staining. Before imaging, the hanging drop chamber was sealed with molten Vaseline. Image acquisition was performed using a Nikon 60×1.2 NA water objective with a *z*-step size of 1 μm.

### BODIPY staining

The lipophilic molecule BODIPY 493/503 (Invitrogen, D3922) was dissolved in 100% DMSO to 1 mg/ml. The working solution of BODIPY was diluted by M9 buffer to 6.7 μg/ml BODIPY (the final concentration of DMSO was 0.8%). The tested animals were picked and incubated in 100 μl of 6.7 μg/ml BODIPY for 30 min and soaked in M9 buffer for 5-10 min until the animals were hydrated and started moving. The recovered animals were then anesthetized with 0.1% tricaine and 0.01% tetramisole in M9 buffer for 15 to 30 min before being transferred to 7% agarose pads for imaging. The diameter of LD was quantified in each oocyte by ImageJ/FIJI.

### CRISPR design, experiments and sequence information

The sequence information of CRISPR design is listed in Table S1. We followed the optimized CRISPR/Cas9 editing protocol that was used in our previous studies (Bai et al., 2020a). The gene-specific guide RNAs were designed with the help of a guide RNA design checker from Integrated DNA Technologies (https://www.idtdna.com/) and were ordered as 4 nmol products from Horizon Discovery (https://horizondiscovery.com/en/dharmacon), along with tracRNA. Repair template design followed the standard protocols (Paix et al., 2015). Young gravid animals (∼20) were injected with the prepared CRISPR/Cas9 injection mix, as described in the literature (Paix et al., 2015; Bai et al., 2020b). The Cas9 protein was purchased from PNA Bio (CP01). All homozygous animals edited by CRISPR/Cas9 were validated by Sanger sequencing. The detailed sequence information for the repair template and guide RNAs are listed in Table S1.

### MIP-MAP and data analysis

Candidate mutations (defined as novel, homozygous and nonsynonymous) were identified by whole-genome sequencing (WGS) as described previously (Smith, 2022). Briefly, sequencing libraries were constructed using the Invitrogen Pure Link Genomic DNA Mini Kit (K1820-01) with genomic DNA from homozygous suppressor-bearing strains. The WGS libraries were pooled and sequenced on a HiSeq 3000 instrument (Illumina) to at least 20-fold coverage. Variants were identified with a pipeline of BBMap (https://sourceforge.net/projects/bbmap/), SAMtools (Li et al., 2009), FreeBayes (Garrison and Marth, 2012 preprint) and ANNOVAR (Wang et al., 2010). Mapping loci for suppressors were identified using molecular inversion probes (MIPs) to single-nucleotide polymorphisms (SNPs), as described previously (Mok et al., 2017). Briefly, suppressor-bearing strains were mated to SNP mapping strain VC20019 (Thompson et al., 2013), engineered via CRISPR to contain the *seip-1(A185P)* mutation. F1 cross-progeny were allowed to self-fertilize, and a minimum of 50 homozygous F2 progeny were pooled for the construction of MIP libraries. SNP allele frequencies were determined using a custom script and plotted with R (http://www.R-project.org) to delimit the mapping interval.

### Data and statistical analyses

The sample size for each experimental group or condition is labeled in the figures and figure legends. Statistical methods and sample numbers are detailed in the corresponding figure legends. Statistical significance for other assays was determined using an unpaired two-tailed *t*-test, a one-way ANOVA with Tukey's post hoc test or a χ^2^ test. ns, not significant; *P<0.05; **P<0.005. Both the Shapiro–Wilk and Kolmogorov–Smirnov normality test indicated that all data follow normal distributions.

This article is part of the collection ‘Translating Multiscale Research in Rare Disease’, which was launched in a dedicated Special Issue edited by Monica Justice, Monkol Lek, Karen Liu and Kate Rauen. See related articles in this collection at https://journals.biologists.com/dmm/collection/39/Rare-Disease.

## Supplementary Material

10.1242/dmm.050524_sup1 Supplementary information
