# Supplementary material for: Identification of genetic suppressors for a BSCL2 lipodystrophy pathogenic variant in Caenorhabditis elegans
Source: Dis Model Mech. 2024 Apr 16;17(6):dmm050524. doi: 10.1242/dmm.050524 (PMC11051982; doi:10.1242/dmm.050524)
Supplement: Supplementary information [file dmm-17-050524-s1.pdf]

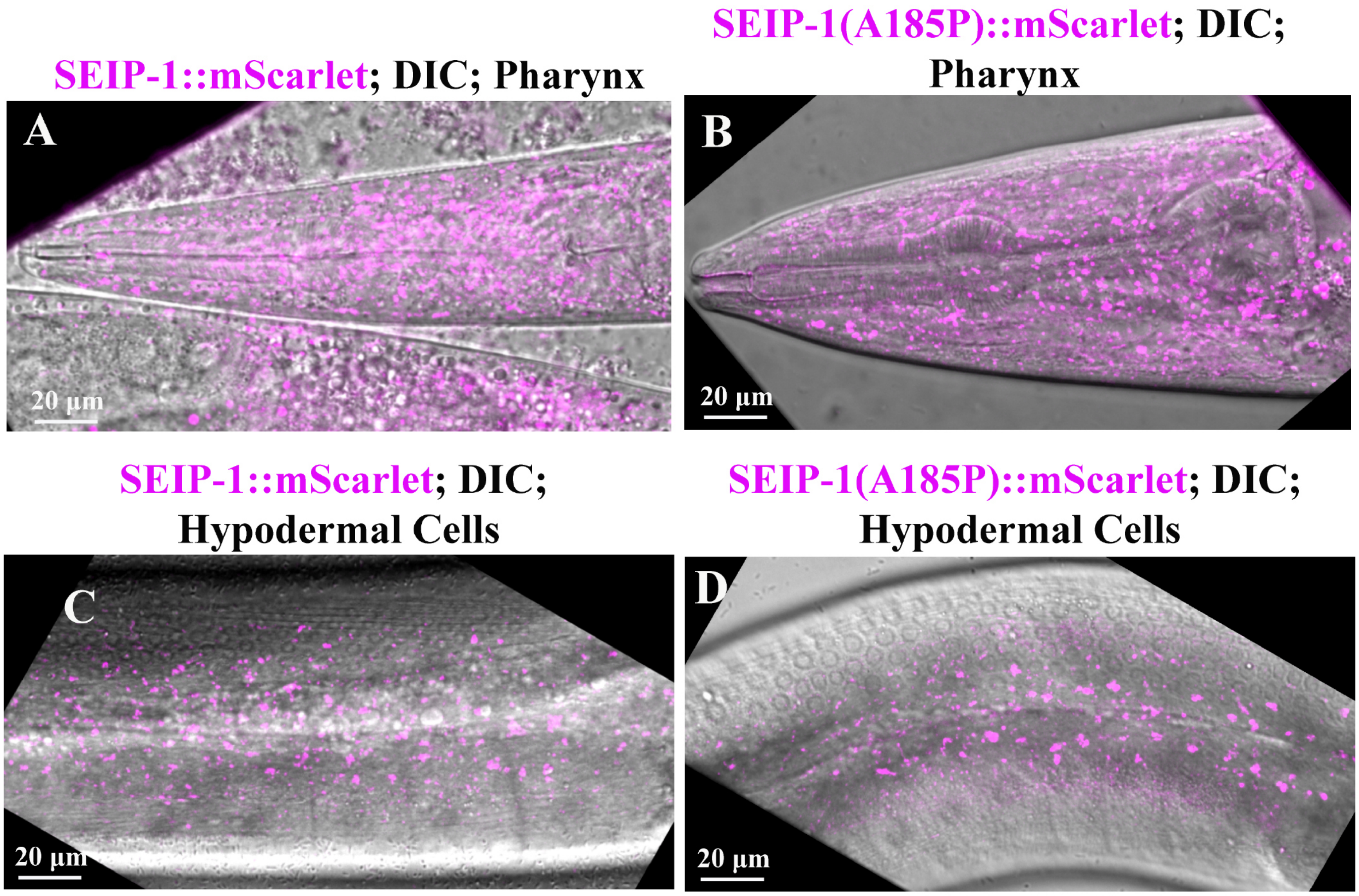

Fig. S1. *seip-1(A185P)::mScarlet* does not disrupt the cellular localization of SEIP-1::mScarlet. (A-L) *seip-1(A185P)::mScarlet* (magenta, A-D) presents an identical expression pattern as *seip-1::mScarlet* only (magenta, A, C) in a variety of cell types, including pharynx (A-B) and hypodermal cells (C-D). DIC images are shown in panels A-D as grey color. Scale bars are indicated in each panel.

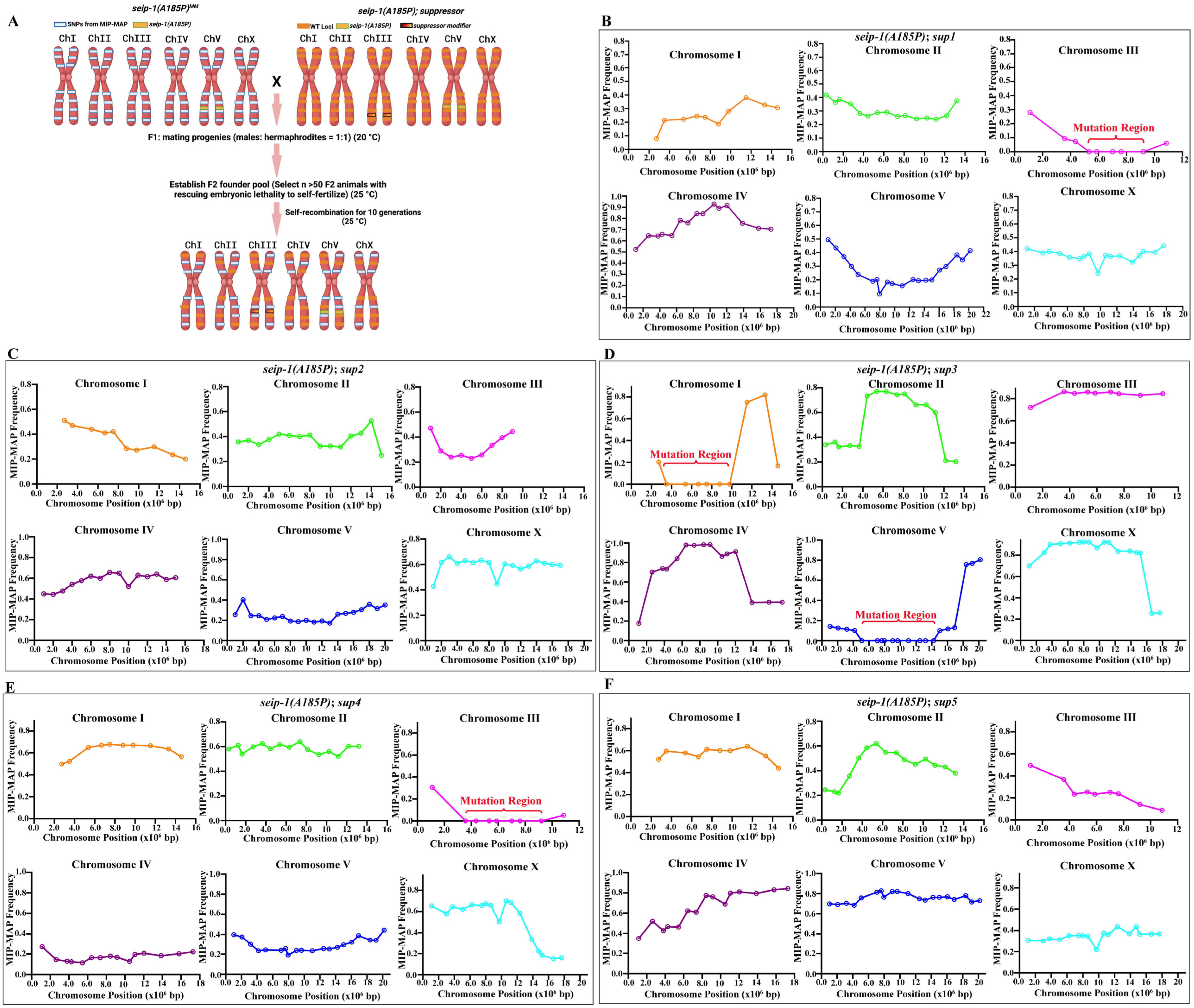

Fig. S2. Mapping of *seip-1(A185P)* suppressors via VC20019 and MIP-MAP sequencing. (A) Diagram of the MIP-MAP workflow to map the SNVs in the suppressor line. Created with BioRender.com. (B-F) The read frequency of the VC20019-specific SNVs across the genome of the pooled F2 progeny from *seip-1(A185P); sup1-5* and *seip-1(A185PMM)* cross. (B, E) An identical candidate mutation-associated interval was identified on Chromosome III in *seip-1(A185P); sup1*, and *seip-1(A185P); sup4*. The strategy graphic was generated with BioRender. com.

Table S1. Sequences for CRISPR design

Strain	Genotype	Description	Sequence Name	Sequence 5'-3'
AG685/ AG751/ AG666	*seip-1(av169[seip-1(A185P)::mScarlet]) V. seip-1(av294[A185P]) V. seip-1(A185P MM) V.*	Generate a point mutation A185P in *seip-1*	crRNA	ACGAGCAGTCACGATTAACT
			Repair Template	TATCAAGCCAAAACGAGCAGTCACGATTAACTCGGGCTCCTCGATctggaaatagagaaataattagagttggac
			Genotyping F1	gaaaattccatccggcatcc
			Genotyping R1	ggcgctgacattaccacata
AG743	*lmbr-1(av288)[P314L] III.*	Generate a point mutation P314L in *lmbr-1*	crRNA	TTGACCACGTCTTCTAGATG
			Repair Template	ACGTTTTCAGTTATATCATTGACCACGTCTTCTAG GTGCAG AGTATCGCTGCGATCAACTTGATGTATCGTTTCA
			Genotyping F1	TCAACGCATCTACGAAGCCA
			Genotyping R1	GAAAGGGATGACCTCTGCCG
AG746	*lmbr-1(av288)[S647F] III.*	Generate a point mutation S647F in *lmbr-1*	crRNA	GTTAACAGCTACTGGGAGAG
			Repair Template	AAAGTTGTCATTCCAACTGTGTTAACAGCTACTGGCAATGCGAATGCAACAACAAGAACTATTGAAGAATTAATAATT
			Genotyping F1	TTTGGGGCTTGCATCGAAAC
			Genotyping R1	aaaaggcgagtagtacccgag
AG750	*lmbr-1(av293[lmbr-1Δ] III.*	Generate a full deletion of *lmbr-1*	crRNA1	CCAGGGCGTATTGGGCCATt
			crRNA2	TGCTTTTCTTATCGCCTCTA
			Repair Template	tctctattgctttttacagatttaataacaccaaA TG TAGGTGATTAGAAAAGCAATTGAatttctttcctttttcaacac
			Genotyping F1	acgaactgtgcctctgtgac
			Genotyping R1	aaaaggcgagtagtacccgag
			Genotyping inner F1	TTTGGGGCTTGCATCGAAAC

Uppercase letters represent the ORF or exon sequence; lowercase letters indicate the sequence of the intron.

Disease Models & Mechanisms • Supplementary information
